# Case of Varicose Vein, Cured by Means of Needles Introduced through the Veins, after the Method Proposed by Davat

**Published:** 1838-04-25

**Authors:** 


### *Case of Varicose Vein, cured by means of needles introduced through the Veins, after the method proposed by Davat.*

George K———, a German, aged 57 years, was admitted into the wards on the 19th of July, 1837, for varicose veins, from which he had suffered for several months. He had had a large ulcer caused by the veins, for which he had been treated in the city by bandages, &c. The ulcer was much reduced in size when he entered, and, after appropriate treatment, healed. On the 12th of August, Dr. NORRIS introduced two acupuncture needles, one behind the vein, and the other through and through it in a line oblique to its axis, and surrounded both by a figure of eight ligatures. Little pain was caused by the operation; the limb was then elevated in a fracture-box; lead water cloths applied, and the antiphlogistic treatment directed.

*August* 15th. The patient complains of no pain, little inflammation has occurred; ligature tightened and treatment continued.

*August* 17th. Slight inflammation at the sutures—same treatment.

*August* 19th. The needles and ligatures were removed, some inflammation around the part, but none to any distance above or below.

*August* 24th. Inflammation increased; slight ulceration at the points where the needles entered; a poultice to the part, and antiphlogistic treatment continued.

*September* 4th. The ulcers have healed; the vein perfectly obstructed; bandages and compress applied along the course of the vein.

*September* 7th. Allowed to walk about; has slight porriginous eruption; treated accordingly.

*September* 15th. Vein obliterated entirely; patient walks without feeling any inconvenience from it.

*September* 17th. Discharged—entirely well.

Within a few weeks the patient was seen, having had no return of his complaint, and continuing constantly at work.

